# Caractéristiques obstétricales liées au cancer du col utérin dans le territoire nord de la région Rabat-Salé-Kenitra, Maroc

**DOI:** 10.11604/pamj.2025.52.102.44900

**Published:** 2025-11-11

**Authors:** Mohamed Zraidi, Nadia Kamel, Ghizelane El Belloute, Khalid Abidi

**Affiliations:** 1 Laboratoire Biologie et Santé, Faculté des Sciences, Université Ibn Tofail, Kenitra, Maroc,; 2 Laboratoire de Pharmacologie, Faculté de Médecine et de Pharmacie, Université Mohamed V, Rabat, Maroc,; 3 Faculté des Sciences Juridiques, Economiques et Sociales, Université Mohamed V, Rabat, Maroc,; 4 Unité de Soins Intensifs Médicaux, Hôpital Universitaire Ibn Sina, Faculté de Médecine et de Pharmacie, Université Mohammed V, Rabat, Maroc

**Keywords:** Cancer du col utérin, caractéristiques obstétricales, région Rabat-Kenitra, Maroc

## Aux éditeurs de Pan African Medical Journal

Le cancer du col utérin (CCU) représente un problème de santé publique à travers le monde, avec 662301 cas de CCU et 348874 décès dus à la maladie en 2022, pour lesquels les génotypes cancérigènes du virus du papillome humain (HPV) sont considérés comme la cause nécessaire [1]. En réponse à ce lourd fardeau évitable, l'Organisation mondiale de la Santé (OMS) a lancé sa stratégie mondiale pour l'élimination du CCU en 2022 soulignant le rôle vital de la vaccination, (le rôle) du dépistage et du traitement contre le HPV et les lésions précancéreuses [1,2]. Il est classé, à l'échelle mondiale, le quatrième cancer chez la femme toutes localisations confondues [3,4]. Au Maroc, l'incidence brute est de 11,6 pour 100000 femmes [5,6]. Le CCU se catégorise parmi les trois principaux cancers touchant les femmes de moins de 45 ans dans 146 des 185 pays [7].

L'objectif de cette étude portée sur un nombre de 297 malades durant la période allant de 2013 à 2021 [8], consiste en l'analyse des caractéristiques obstétricales liées à ce cancer à savoir: la gestité, la parité, l'âge de la première grossesse ou l'âge du premier rapport sexuel, menstruations et enfin la notion de prise de contraception orale, des patientes atteintes de lésions précancéreuses ou du CCU dans le territoire nord de la région Rabat-Salé-Kenitra au Maroc (Gharb du Maroc). Les méthodes statistiques utilisées sont l'analyse de variance ANOVA à plan factoriel, l'analyse des coefficients de corrélation (r), le coefficient de détermination (r^2^) et le test khi^2^.

En effet, une gestité moyenne est de 4,81±0,16 grossesses avec un minimum de 0 grossesse (femme nullipare) et un maximum de 15 grossesses (distribution gaussienne, coefficient d'asymétrie=0,78 et aplatissement =0,73). La parité moyenne est de 4,22±0,14, avec un minimum de 0 (femme nullipare) et un maximum de 15 (distribution gaussienne, coefficient d'asymétrie=0,85 et aplatissement =1,60). Le Tableau 1 présente la distribution des patientes selon la parité et il en ressort que 64,3% sont des patientes multipares (3 à 6 pares) et 14,1% sont des grandes multipares (≥ 7 parités).

**Tableau 1:** répartition des patientes selon la parité

Parité	Nombre	Pourcentage
Grande multipare (≥ 7 pares)	39	14,1
Multipares (3-6 pares)	178	64,3%
Secondipares (2 pares)	28	10,1%
Primipares	23	8,3%
Nullipares	9	3,2%

Les résultats de l'ANOVA de l'âge en fonction de la gestité et de la parité des patientes. En effet, le test effectué sur les données révèle une disparité remarquable entre les valeurs moyennes de la gestité des différentes catégories d'âge (F=15,68; p<0,000) de même pour la parité (F=13,72; p<0,000). Toutefois, une forte corrélation positive associe la gestité, la parité et l'âge (p<0,000). Concernant la répartition des patientes selon l'âge à la première grossesse, en l'occurrence l'âge du premier rapport sexuel, elle montre que l'âge moyen est de 20,47±0,361 ans (minimum = 13 ans; maximum = 39), avec une médiane de 19 ans. Concernant les résultats du test khi^2^ entre le lieu de résidence et l'âge à la première grossesse, une liaison significative associe ces deux facteurs (khi^2^ = 9,04; p<0,05). Par ailleurs, plus de 54% des patientes ayant eu leur première grossesse avant 20 ans proviennent du rural et dont 11% des patientes l'ont eue avant 15 ans. Cependant, 46% des patientes ayant eu leur première grossesse entre 20 et 30 ans sont d'origine urbaine.

Le Tableau 2 montre les résultats de l'ANOVA «effet de l'âge de la première grossesse » sur la répartition du nombre de grossesses et de parités. Le test de Fisher indique une association remarquable entre les valeurs moyennes (p<0,000). Une corrélation négative très hautement significative associe l'âge de la première grossesse et la gestité (r = -0,4; p<0,000). D'un autre côté, une corrélation négative très hautement significative associe l'âge de la première grossesse et la parité (r=-0,36; p<0,000). Concernant la répartition des patientes selon le cycle menstruel, les résultats de l'ANOVA « Effet de cycle menstruel » sur la répartition du nombre de grossesses et de parités et le test de Fisher montrent une association très hautement significative avec respectivement p<0,016 et p<0,024.

**Tableau 2:** ANOVA « effet âge première grossesse » sur la répartition de la gestité et la parité

Age première grossesse	N	Gestité		Parité	
		Moyenne	Fisher	Moyenne	Fisher
Moins de 15	17	6,12(c)	7,75*** (p<0,000)	4,82(c)	6,39*** (p<0,000)
Entre 15 et 20	108	4,79(bc)		4,34(bc)	
Entre 20 et 25	39	4,28(bc)		3,64(abc)	
Entre 25 et 30	21	3,19(ab)		2,76(ab)	
Supérieur à 30	7	2,14(a)		2,00(a)	
Total	192	4,53		3,98	

Par contre, la répartition des malades en fonction de la prise de contraception hormonale montre que 73,2% répondent « oui » alors que 26,8% disent qu'elles n'utilisaient jamais la contraception hormonale. La distribution s'est montrée gaussienne (coefficient d'asymétrie = 0,319 et un coefficient d'aplatissement = 0,82). D'un autre côté, le test khi^2^ indique une importante liaison entre la situation matrimoniale et la prise de la contraception hormonale (khi^2^ = 9,78; p<0,002). Le risque attribuable (RA) est de 21,84%. Enfin, d'après l'analyse globale par ACP des variables (âge; âge de la première grossesse; la durée de la prise de contraception, la gestité et la parité), deux groupes distincts ont été reformulés qui évoluent dans le sens contraire.

## Conclusion

Le CCU est multifactoriel et la valeur ajoutée de notre travail est l'identification du profil des femmes à risque élevé du CCU afin de les dépister précocement. Le Maroc connait un progrès considérable dans la prévention [9], en particulier la prévention primaire, tout en sachant qu'un schéma vaccinal d'une dose (au lieu de deux) a été optimisé pour les filles scolarisées âgées de 11 ans [10]. Enfin, certaines recommandations devraient être soulignées, en se basant essentiellement sur la promotion de la vaccination anti-HPV par une communication bien menée pour lutter contre les réticences, essentiellement les réticences créées par les rumeurs du « complot vaccinal » surtout après la pandémie de COVID-19.

## Remerciements

Nous remercions tous ceux qui ont participé à ce travail.
